# Overexpression of *OsLCT2*, a Low-Affinity Cation Transporter Gene, Reduces Cadmium Accumulation in Shoots and Grains of Rice

**DOI:** 10.1186/s12284-021-00530-8

**Published:** 2021-10-24

**Authors:** Li Tang, Jiayu Dong, Longtao Tan, Zhongying Ji, Yaokui Li, Yuantao Sun, Caiyan Chen, Qiming Lv, Bigang Mao, Yuanyi Hu, Bingran Zhao

**Affiliations:** 1 State Key Laboratory of Hybrid Rice, Hunan Hybrid Rice Research Center, Changsha, 410125 China; 2 Longping Branch of Graduate School, Hunan University, Changsha, 410125 China; 3 Key Laboratory of Agro-Ecological Processes in Subtropical Region, Institute of Subtropical Agriculture, Chinese Academy of Sciences, Changsha, 410125 China

**Keywords:** Cadmium, OsLCT2, Rice, Root-to-shoot translocation, Overexpression, Metal transporter

## Abstract

Cadmium (Cd)-contaminated rice is a serious issue affecting food safety. Understanding the molecular regulatory mechanisms of Cd accumulation in rice grains is crucial to minimizing Cd concentrations in grains. We identified a member of the low-affinity cation transporter family, OsLCT2 in rice. It was a membrane protein. *OsLCT2* was expressed in all tissues of the elongation and maturation zones in roots, with the strongest expression in pericycle and stele cells adjacent to the xylem. When grown in Cd-contaminated paddy soils, rice plants overexpressing *OsLCT2* significantly reduced Cd concentrations in the straw and grains. Hydroponic experiment demonstrated its overexpression decreased the rate of Cd translocation from roots to shoots, and reduced Cd concentrations in xylem sap and in shoots of rice. Moreover, its overexpression increased Zn concentrations in roots by up-regulating the expression of *OsZIP9*, a gene responsible for Zn uptake. Overexpression of *OsLCT2* reduces Cd accumulation in rice shoots and grains by limiting the amounts of Cd loaded into the xylem and restricting Cd translocation from roots to shoots of rice. Thus, *OsLCT2* is a promising genetic resource to be engineered to reduce Cd accumulation in rice grains.

**Supplementary Information:**

The online version contains supplementary material available at 10.1186/s12284-021-00530-8.

## Introduction

In recent years, the widespread contamination of farmland by toxic heavy metals has become an environmental problem, raising concerns about health risks to people all over the world. Cadmium (Cd) is one of the most toxic heavy metals to organisms. It has a long biological half-life (10–30 years) in the human body and is associated with various diseases, including renal damage, osteoporosis, and cancers (Godt et al. 2006; Uraguchi and Fujiwara 2013; Clemens and Ma 2016). Rice is the principal food source for over half of the global population and is prone to accumulate more Cd than other cereal crops (Zhao and Wang 2019). Chronic dietary consumption of rice with excessive Cd is the major source of human Cd-intake for local residents of Cd-contaminated areas (Hu et al. 2016). About 90% of rice is produced in Asia, and Cd contamination of rice has been reported in many Asian countries (Hu et al. 2016; Tang et al. 2017). Therefore, it is important and urgent to minimize Cd accumulation in rice grains for food safety and human health. Screening and breeding of rice cultivars with low Cd concentrations in the grains is regarded as the most cost-effective and eco-friendly strategy (Chi et al. 2018).

Understanding the physiological and molecular mechanisms of Cd uptake, translocation, and distribution in rice is critical for developing rice cultivars with low Cd levels. Following Cd uptake from the soil by roots, Cd may be sequestered into the vacuoles of root cells or translocated from roots to shoots via xylem and then distributed/redistributed to different organs via nodes (Clemens and Ma 2016). The uptake and transport of Cd is mediated by transporters of divalent metals, such as manganese (Mn), iron (Fe), zinc (Zn), and calcium (Ca), which show chemical characteristics similar to those of Cd. To date, several transporter families have been reported involved in this process in rice, including the natural resistance-associated macrophage protein (NRAMP) family, the zinc-regulated/iron-regulated transporter-like protein (ZIP) family, and heavy metal ATPase (HMA) family.

OsNRAMP5 is the major transporter responsible for the uptake of both Cd and Mn, and the knockout of *OsNRAMP5* dramatically reduces Cd and Mn concentrations in rice (Ishikawa et al. 2012; Sasaki et al. 2012). Mutation of *OsNRAMP5* via the CRISPR/Cas9 system produced a low Cd-accumulating *indica* hybrid rice without compromising its grain yield normally (Tang et al. 2017). However, *osnramp5* mutants exhibited impaired growth under conditions of low Mn supply caused by Mn deficiency in plants (Yang et al. 2014). In addition, iron-regulated transporters OsIRT1 and OsIRT2 and Mn transporters OsNRAMP1 and OsCd1 (a member of a major facilitator family) also participate in Cd uptake in rice, but their contributions are relatively minor compared to that of OsNRAMP5 (Nakanishi et al. 2006; Senoura et al. 2011; Yan et al. 2019; Chang et al. 2020).

Once Cd enters into the roots, it moves radially into the stele for xylem loading and then transfers to shoots mainly via the xylem. In the process of radial transport of Cd in roots, chelation, compartmentalization, and adsorption retain most of the Cd in roots and consequently restrict Cd translocation from roots to shoots (Nocito et al. 2011). OsHMA3 is localized to the tonoplast of root cells and compartmentalizes Cd into vacuoles, thus limiting the quantities of Cd loaded into the xylem (Ueno et al. 2010; Miyadate et al. 2011; Sasaki et al. 2014). Previous studies have identified two loss-of-function alleles of *OsHMA3* harboring a substitution at amino acid position 80 or 380 and one weak allele of *OsHMA3* carrying an amino acid mutation at position 512. Rice varieties with the above alleles of *OsHMA3* had high Cd concentrations in the shoots and grains of rice due to a weakened ability to sequester Cd in the root vacuoles (Ueno et al. 2010; Yan et al. 2016; Sun et al. 2019).

Several transporters, including OsHMA2, OsZIP7, and cation/Ca exchanger 2 (OsCCX2), have been reported to facilitate the root-to-shoot translocation of Cd and delivery of Cd to developing tissues and grains (Takahashi et al. 2012; Yamaji et al. 2013; Hao et al. 2018; Tan et al. 2019). All of them are not Cd-specific transporters. Among them, OsHMA2 and OsZIP7 showed transport activity of Zn and Cd, and OsCCX2 showed efflux transport activity of Ca and Cd. Knockout of *OsHMA2* or *OsZIP7* resulted in severe reductions of grain yield and was mainly attributed to Zn deficiency in the plants (Yamaji et al. 2013; Tan et al. 2019). Knockout of *OsCCX2* reduced the 1000-grain weight, which was attributed to decreased Ca transport (Hao et al. 2018). A defensin-like protein in rice, OsCAL1, can chelate Cd and assist Cd secretion to apoplastic spaces, and consequently accelerates long-distance Cd transport via the xylem (Luo et al. 2018). Knockout of *OsCAL1* reduced Cd translocation to shoots, but it did not affect Cd concentrations in the grains of rice (Luo et al. 2018).

Cd can be delivered to grains via phloem transport, which is regulated by OsHMA2 and low-affinity cation transporter 1 (OsLCT1). OsHMA2 is implicated in reloading Zn and Cd from the parenchyma tissues into the phloem of diffuse vascular bundles (Yamaji et al. 2013). OsLCT1 is the only member of the LCT family in rice that has been identified and functionally characterized. OsLCT1 is a plasma membrane-localized efflux transporter of Cd, potassium (K), Ca, magnesium (Mg) and Mn. In the uppermost node, *OsLCT1* was expressed around the enlarged vascular bundles and diffused vascular bundles. Knock down of *OsLCT1* by RNAi did not affect the Cd concentration in xylem sap, but it did decrease the Cd concentration in phloem sap, strongly suggesting that OsLCT1 is involved in xylem-to-phloem transfer of Cd. These RNAi lines also showed about a 50% reduction of Cd in grains without affecting mineral nutrient concentrations and plant growth (Uraguchi et al. 2011).

Compartmentalization of metal in the vacuole, endoplasmic reticulum (ER), or Golgi apparatus by endomembrane-localized transporters is crucial for plant tolerance to the stress of excess metal (Shao et al. 2017; De Caroli et al. 2020). However, OsHMA3 is the only identified intracellular transporter that sequesters Cd in the vacuoles of roots in rice. The functions and molecular mechanisms of endomembrane-localized Cd transporters in rice are poorly understood. In this study, we cloned *OsLCT2*, a novel member of the *LCT* family in rice, and found that its GFP-fusion protein was localized to the ER when transiently expressed by the 35S promoter. Overexpression of *OsLCT2* weakened Cd translocation from roots to shoots and consequently reduced Cd concentrations in rice shoots and grains.

## Results

### Cloning and Characterization of *OsLCT2*

Rice OsLCT1 is an important Cd transporter (Uraguchi et al. 2011, 2014). Unexpectedly, no *OsLCT1* fragments could be obtained by PCR amplification from the DNA of *indica* rice cultivars, Huazhan, 93-11 and Shuhui 498. Hence, BLAST searches were conducted using the amino acid sequences of *OsLCT1* against the genome of the rice cultivar Shuhui498 in the National Center for Biotechnology Information (NCBI) database. The *OsLCT1* sequence could not be aligned, confirming the absence of *OsLCT1* in the genome of Shuhui498. However, we found a putative rice LCT protein, designated as OsLCT2. Because the full-length cDNA sequence of *OsLCT2* was not available in the Rice Annotation Project Database (http://rapdb.dna.affrc.go.jp/), we performed 5′- and 3′- rapid amplification of cDNA ends (RACE) to clone the full-length cDNA of *OsLCT2* (GenBank accession number: MW757982) from the *indica* rice cultivar Huazhan.

**Fig. 1:**
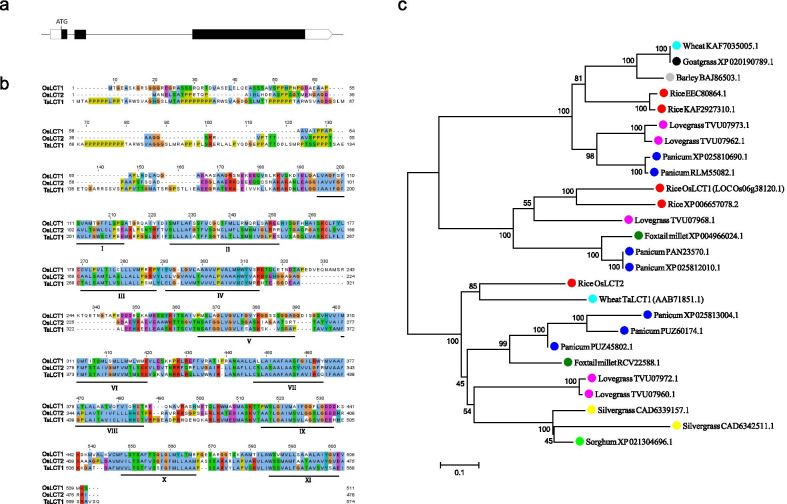
Gene structure of *OsLCT2* and phylogenic relationships of LCT proteins. **a** Schematic diagram of *OsLCT2* gene structure. UTRs, exons, and introns are indicated by blank rectangles, black rectangles, and black lines, respectively. **b** Alignment of the predicted amino acid sequences of OsLCT1, OsLCT2 and TaLCT1. Identical or similar amino acids are indicated by the same background color. Non-conservative amino acids are indicated by different background colors. The predicted trans-membrane domains are underlined and labeled by Roman numerals. **c** Phylogenic tree of LCT-like proteins. The predicted LCT amino acid sequences were used to construct the phylogenetic tree using MEGA7. Unidentified proteins are represented by accession numbers in the NCBI database. The scale bar of 0.1 is equal to 10% sequence divergence

*OsLCT2* contains three exons and two introns (Fig. 1a), and encodes a peptide of 478 amino acids. It is located at position 23,774,023 to 23,776,721 on chromosome 6 in the Shuhui498 genome. Similar to TaLCT1 and OsLCT1, OsLCT2 has a hydrophilic amino N terminus containing sequences enriched in Pro, Ser, Thr, and Glu, collectively known as the PEST sequence (Fig. 1b) (Kennedy and Rinne 1997). Both the TMPRED and CCTOP programs predicted that OsLCT2 is a membrane protein with eleven transmembrane domains (Fig. 1b). Phylogenetic analysis showed that LCT-like proteins were only found in grass plants (Fig. 1c). Among those LCT members in grass plants, the closest homolog of OsLCT2 was TaLCT1, which also belonged to the same subgroup as OsLCT2 (Fig. 1c). Amino acid sequence alignment revealed that OsLCT2 shared 55% and 33% sequence identity with TaLCT1 and OsLCT1, respectively. To explore the evolutionary relationship between OsLCT2 and OsLCT1, phylogenetic analysis was conducted using their predicted amino acid sequences and LCT-like proteins in eight wild *Oryza* species. OsLCT2 and its orthologs in *Oryza rufipogon* were in the same clade with 98.5% sequence identity, whereas the OsLCT1 showed high similarity to the LCT-like proteins in *Oryza barthii*, *Oryza glaberrima*, and *Oryza nivara*, with 98.6%, 98.6%, and 95.7% sequence identity, respectively (Additional file 1: Fig. S1a)*.* These results revealed that orthologs of both OsLCT2 and OsLCT1 existed in wild rice species, and the considerable sequence divergence between OsLCT2 and OsLCT1 derived from the divergence of their ancestor gene pools. This finding implied that they diverged before the diversification of *Oryza* genus.

We further analyzed natural variations of *OsLCT2* coding sequence (CDS) using 3,620 rice accessions of the MBKbase-rice database (Peng et al. 2020). Twelve single-nucleotide polymorphisms (SNPs) were detected in the *OsLCT2* CDS region (Additional file 1: Fig. S1b). Based on these variations, its CDS region was classified into nine haplotypes (rare haplotypes of < 10 accessions were not shown). Haplotype 1, which was harbored in Huazhan and Shuhui 498, was also prevalent in the four main rice subpopulations, accounting for 68.3% of *indica*, 43.3% of *temperate japonica*, 72.3% of *tropical japonica*, and 39.6% of *aus* (Additional file 1: Fig. S1b, c). Notably, there were different proportions of *OsLCT2* deletions in these rice subpopulations. *OsLCT2* was absent in about 54% of *temperate japonica* and *aus*, but only in a small fraction of *indica* (4.3%) and *tropical japonica* (14.3%) (Additional file 1: Fig. S1b, c).

### Expression Pattern of *OsLCT2*

**Fig. 2:**
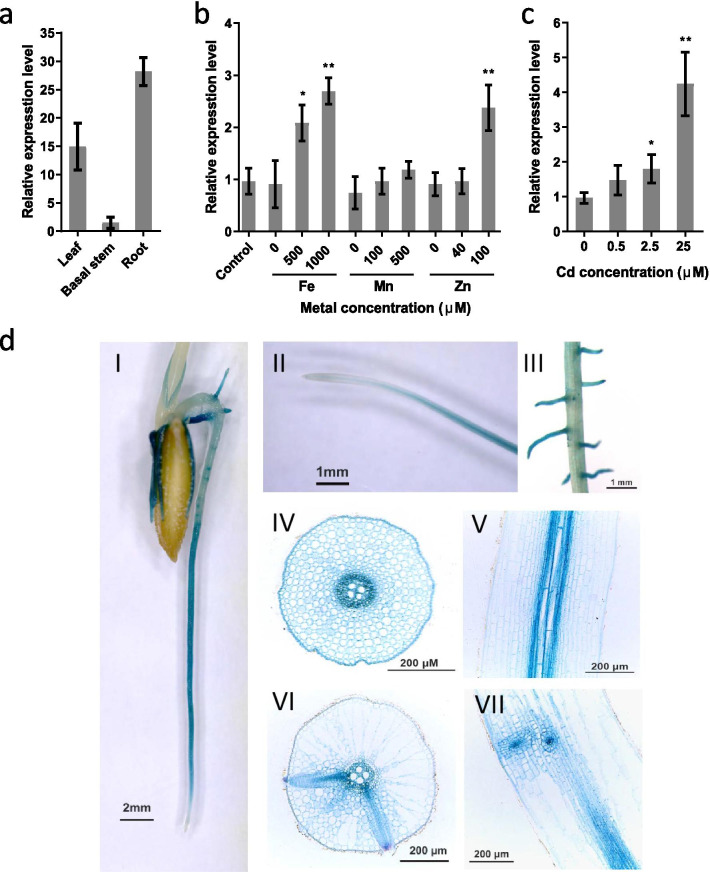
Expression patterns of *OsLCT2*. **a**–**c** The expression of *OsLCT2* was determined by qRT-PCR. **a** Tissue-dependent expression of *OsLCT2* in 2-week-old seedlings. The leaf, root and basal stem (2 cm above the roots) were sampled for expression analysis. Relative expression of *OsLCT2* is shown, with its expression in the basal stem set to 1. **b** Response of *OsLCT2* in seedling roots to the absence or excess of Fe, Mn or Zn. Seven-day-old seedlings were grown in Yoshida nutrient solution without Fe, Mn, or Zn or with high concentrations of Fe, Mn, or Zn for 7 d. Seedlings grown in standard Yoshida nutrient solution were used as controls. **c** Response of *OsLCT2* in seedling roots to external Cd. Two-week-old seedlings were exposed to different concentrations of Cd for 24 h. Data show means ± SD of three biological replicates. In **b** and **c**, asterisks above the bars indicate significant differences between the treatments and controls (*P < 0.05, **P < 0.01; Student’s *t*-test). **d** Histochemical GUS staining of roots of transgenic rice plants transformed with *OsLCT2* promoter-driven *GUS* constructs. Tissue-specific expression of *OsLCT2* in the entire roots (I), root tip (II), lateral roots (III), root elongation zone (IV, V), and maturation zone (VI, VII) visualized in cross section (IV, VI) and vertical section (V, VII)

The expression profiles of *OsLCT2* in rice seedlings were investigated by quantitative real-time PCR (qRT-PCR). The expression level of *OsLCT2* in roots was relatively high, followed by leaves, with the lowest expression level in the basal region (2 cm above the roots) (Fig. 2a). To investigate the effects of metal deficiency and excess on the expression of *OsLCT2*, rice plants were grown in nutrient solutions either free of Fe, Mn, or Zn or containing high concentrations of Fe, Mn, or Zn for 7 d. Expression of *OsLCT2* was unaffected by the deficiency of those metals, while it was induced by excess Fe and Zn (Fig. 2b). To determine the responses of *OsLCT2* to Cd stress, rice seedlings were treated with 0, 0.5, 2.5 or 25 μM Cd for 24 h. The *OsLCT2* expression in roots was induced by Cd in a dose-dependent manner. Its transcripts increased by about fourfold in response to 25 μM Cd, but slightly increased in response to 2.5 μM Cd concentrations when compared with that in the absence of Cd (Fig. 2c).

To analyze the tissue specificity of *OsLCT2* expression, we determined *OsLCT2* promoter activity by assessing β-glucuronidase (GUS) activity histochemically in transgenic rice plants expressing the *GUS* gene driven by the *OsLCT2* promoter. The GUS staining was mainly observed in the elongation and maturation zones of the root but not in the meristematic zone and root cap (Fig. 2d I, II). In the cross sections and vertical sections of the elongation and maturation zone of the primary root, GUS activity appeared in all the tissues, including epidermis, cortex and stele, with the strongest signal in pericycle and stele cells adjacent to the xylem (Fig. 2d IV–VII). As the primary root matured, the GUS staining faded away, except for a pronounced staining in the lateral roots (Fig. 2d I, III). The observations in the cross sections and vertical sections of the maturation zone where the lateral roots began to form showed that GUS signal occurred mainly in the stele of the primary and lateral roots, especially the undifferentiated vascular tissue of the lateral roots (Fig. 2d VI–VII).

### Subcellular Localization of OsLCT2

**Fig. 3:**
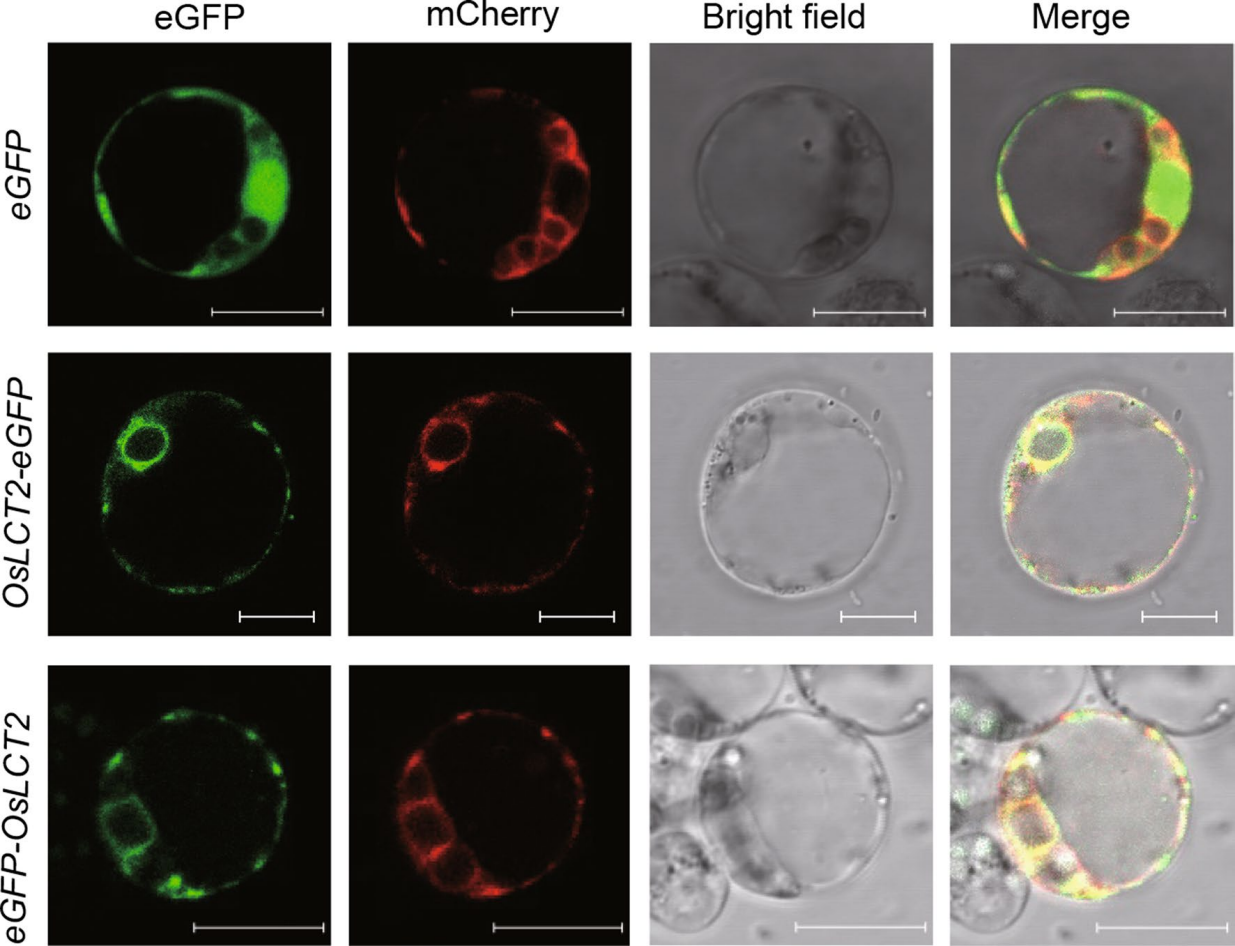
Subcellular localization of OsLCT2. *eGFP*, *OsLCT2-eGFP* or *OsLCT2-eGFP* was co-expressed with *HDEL-mCherry*, an endoplasmic reticulum localization marker, in rice protoplasts. The upper panels show the localization of eGFP and HDEL-mCherry as controls. The middle panels show the localization of OsLCT2-eGFP and HDEL-mCherry. The lower panels show the localization of eGFP- OsLCT2 and HDEL-mCherry. Scale bar, 10 μm

To detect the subcellular localization of the OsLCT2 protein, OsLCT2 was separately fused to the N-terminus or C-terminus of an enhanced GFP (eGFP) via a 16 amino-acid linker. *OsLCT2-eGFP* or *eGFP-OsLCT2* driven by the constitutive cauliflower mosaic virus 35S promoter were cloned into a transient expression vector and introduced into rice leaf protoplasts. Both OsLCT2-eGFP and eGFP-OsLCT2 fusion proteins were observed at the ER, as evidenced by their fluorescence signals overlapping with the ER retention signal His-Asp-Glu-Leu (HDEL) fused with the red fluorescent protein mCherry (Fig. 3). In contrast, the control vector (containing *eGFP* alone driven by the 35S promoter) produced green fluorescence in the cytosol and nucleus (Fig. 3).

### Overexpression of *OsLCT2* Reduces Cd Concentration in Rice Grains

**Fig. 4:**
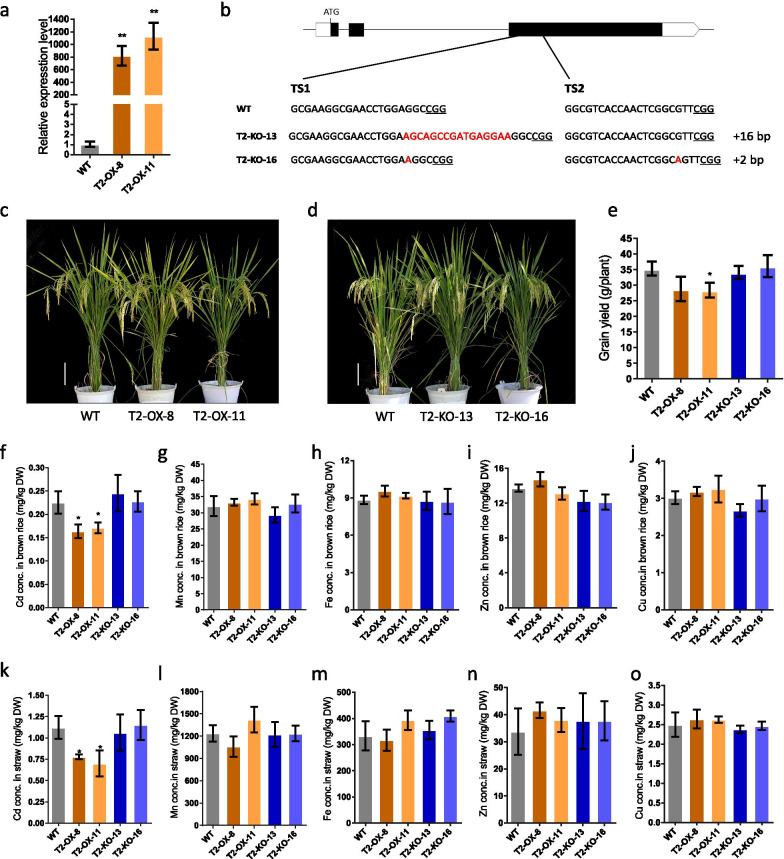
Phenotypic and metal analysis of *OsLCT2*-transgenic lines and the wild-type (WT) grown in a paddy field. **a** Expression levels of *OsLCT2* in roots of overexpression lines relative to that in the WT by qRT-PCR. **b** Structure of *OsLCT2* and mutated sequences of *OsLCT2* knockout lines. The differences in target nucleotide sequence between the WT and two mutant alleles are highlighted in red. To the right of the sequence, ‘+’ signs indicate the number of nucleotides inserted. **c**, **d** Phenotypes of the WT and overexpression lines (**c**) and knockout lines (**d**) at the maturity stage. Scale bar, 18 cm. **e** Grain yield of transgenic lines. **f**–**j** The concentrations of Cd (**f**), Mn (**g**), Fe (**h**), Zn (**i**) and Cu (**j**) in brown rice of transgenic lines. **k**–**o** The concentrations of Cd (**k**), Mn (**l**), Fe (**m**), Zn (**n**) and Cu (**o**) in the straw of transgenic lines. Data show means ± SD of three biological replicates. One asterisk above bars indicates significant difference from the WT at *P* < 0.05 (Student’s *t*-test). DW, dry weight

To investigate the physiological role of *OsLCT2* in rice, we generated two independent overexpression lines driven by a maize ubiquitin promoter and two independent knockout lines using CRISPR/Cas9 technology. The two overexpression lines greatly enhanced expression of *OsLCT2*, compared with that of wild-type (WT) plants, according to the qRT-PCR analysis (Fig. 4a). Sequencing confirmed that each knockout line had insertions that caused a frameshift of the coding sequence (Fig. 4b).

When plants were grown in a field contaminated with 0.65 mg/kg Cd, overexpressing *OsLCT2* tended to reduce grain yield compared with that of the WT, although the difference was only statistically significant in one overexpression line (Fig. 4c, e; Additional file 1: Fig. S2). In contrast, no apparent changes of morphological phenotype or grain yield were found between the knockout lines and the WT (Fig. 4d, e). We then measured Cd, Mn, Fe, Zn and Cu accumulation in these plants at the maturity stage to evaluate whether *OsLCT2* affected metal accumulation in plants grown in field conditions. The Cd concentrations in the brown rice and straw of the overexpression lines were significantly lower by 24.1–27.5% and 30.7–37.5%, respectively, compared with those of the WT (Fig. 4f, k). These results suggest that *OsLCT2* overexpression may not only affect Cd in the grain but also in all above-ground tissues. However, the Mn, Fe, Zn and Cu concentrations in brown rice and straw were similar between the overexpression lines and WT (Fig. 4g–j, l–o). In comparison, all of the above metal concentrations in brown rice and straw did not differ significantly between the knockout lines and the WT (Fig. 4f–o).

### Overexpression of *OsLCT2* Weakens Root-to-Shoot Translocation of Cd, Mn, Fe and Zn

**Fig. 5:**
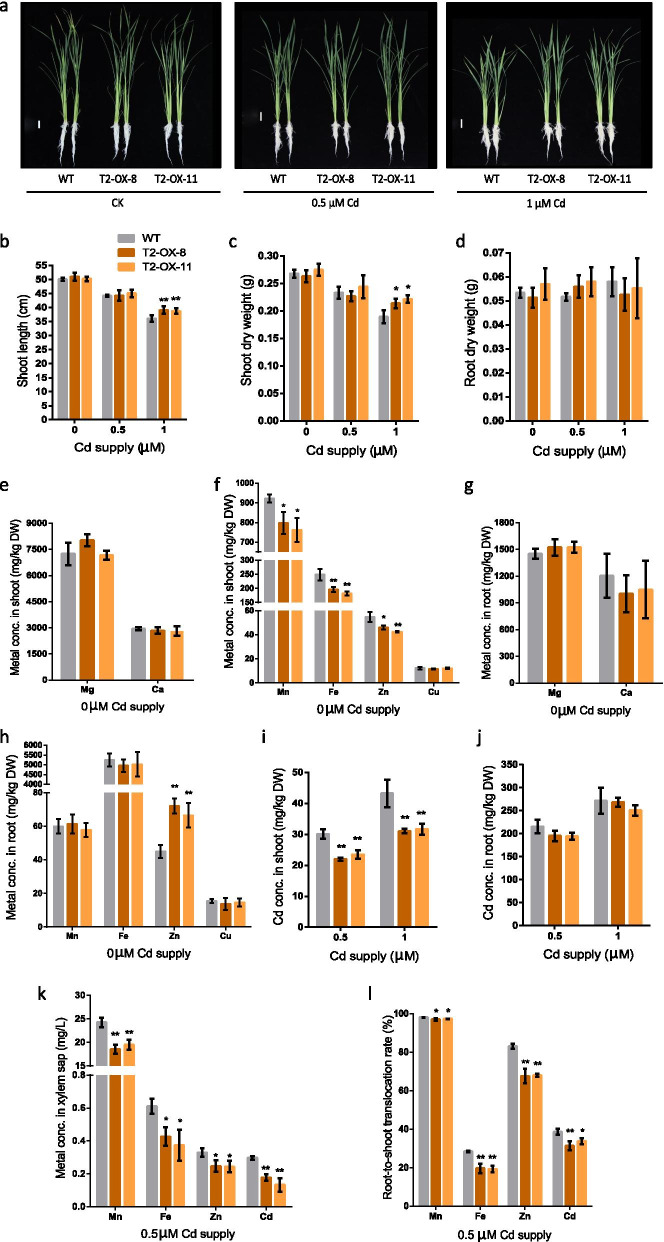
Phenotypic and metal analysis of *OsLCT2* overexpression lines and the WT grown hydroponically. Two-week-old seedlings grown hydroponically were exposed to 0, 0.5 or 1 μM Cd for two weeks. **a** Phenotypes of the WT and overexpression lines. Scale bar, 3 cm. **b**–**d** Shoot length (**b**), shoot dry weight (**c**) and root dry weight (**d**) of overexpression lines and the WT. **e**–**h** Metal concentrations in shoots (**e, f**) and roots (**g, h**) of overexpression lines and the WT without Cd treatment. **i**, **j** Concentrations of Cd in shoots (**i**) and roots (**j**) of overexpression lines and the WT at 0.5 or 1 μM Cd supply. **k**, **l** Metal concentrations in xylem sap (**k**) and metal translocation rates from roots to shoot (**l**) of overexpression lines and the WT at 0.5 μM Cd supply. Data show means ± SD of three biological replicates. Asterisks above the bars indicate significant differences from the WT (*P < 0.05, **P < 0.01; Student’s *t*-test). DW, dry weight

To understand the cause of *OsLCT2* overexpression reducing Cd concentrations in rice straw and grains, two-week-old seedlings of hydroponically-grown *OsLCT2* overexpression lines and WT plants were transferred into nutrient solutions containing 0, 0.5 or 1 μM Cd and kept in solutions for 14 days. Growth responses and divalent metal concentrations in shoots and roots of the overexpression lines and WT were investigated. *OsLCT2* overexpression lines and WT plants showed comparable growth within each of the 0 and 0.5 μM Cd treatments, while shoots of overexpression lines were 7–8% longer and 13–17% heavier (dry weight) in the 1 μM Cd treatment (Fig. 5a–d). The results suggest that overexpressing *OsLCT2* does not affect seedling growth and enhances rice tolerance to excess Cd under hydroponic culture with sufficient mineral nutrition.

Analysis of metal concentrations showed that there were no differences in Mg, Ca and Cu concentrations in both the shoots and roots between the overexpression lines and WT plants (Fig. 5e–h), while the Mn, Fe, Zn concentrations in shoots were significantly lower in the overexpression lines than in the WT with and without Cd stress (Fig. 5f; Additional file 1: Fig. S3a–c). The Mn and Fe concentrations in roots were similar between the WT plants and overexpressing lines, whereas the Zn concentration in roots was significantly higher in the overexpression lines than in the WT plants with or without Cd treatment (Fig. 5h; Additional file 1: Fig. S3d, e). Under hydroponic conditions without Cd, growth of the overexpression lines was similar to that of the WT plants (Fig. 5a), suggesting that the reduced concentrations of Mn, Fe, and Zn in shoots were sufficient for the normal growth and development of overexpression lines.

In the presence of 0.5 and 1 μM Cd, *OsLCT2* overexpression lines exhibited 21.8–26.9% and 26.7–28.1% lower Cd concentrations in shoots, respectively, but similar Cd concentrations in roots compared to those of the WT (Fig. 5i, j). In the presence of 1 μM Cd, overexpression lines showed better Cd tolerance than the WT, which might be attributed to the lower Cd concentrations in shoots that reduced the inhibitory effect of Cd on growth of plants (Fig. 5a). Besides, the overexpression lines showed lower Cd, Mn, Fe and Zn concentrations in the xylem sap than that of the WT (Fig. 5k). As a result, its overexpression significantly decreased the percentages of Cd, Mn, Fe and Zn translocated from roots to shoots (Fig. 5l). Among them, the proportion of Mn transported to the shoots was slightly lower, while the proportions of Cd, Zn and Fe transported to the shoots were moderately lower than those of the WT (Fig. 5l). These results indicate that *OsLCT2* has poor ability to discriminate among the divalent metal cations Cd, Mn, Fe and Zn. Its overexpression weakens the translocation of these four metals from roots to shoots by limiting their amounts loaded into the xylem.

### Effect of *OsLCT2* Overexpression on Expression of *OsZIP* Genes

**Fig. 6:**
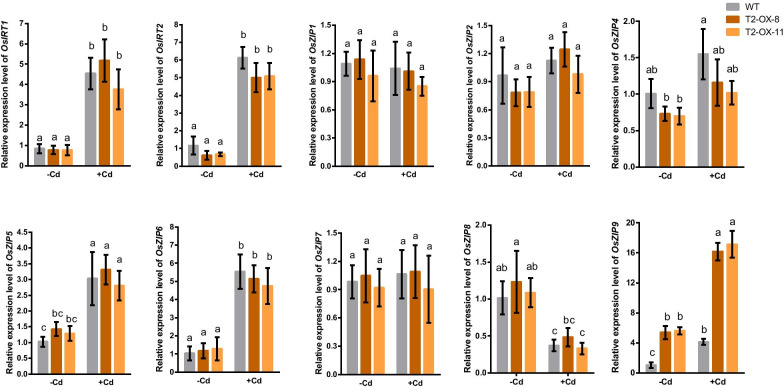
Expression of ten *OsZIP* genes in roots of *OsLCT2* overexpression lines and the WT. Seedlings were exposed to 0 or 0.5 μM Cd for 24 h. Expression levels were determined by qRT-PCR. Expression levels relative to WT without Cd treatment are shown as means ± SD of three biological replicates. Different letters above the bars indicate significant differences at *P* < 0.05 (one-way ANOVA, Tukey’s test)

Because the ZIP family displays transport activity of various cations, including Zn, Fe, Mn and Cd (Lee and An 2009; Ajeesh Krishna et al. 2020), we examined expression of ten genes belonging to the *OsZIP* family in roots of *OsLCT2* overexpression lines and the WT in the presence or absence of 0.5 μM Cd for 24 h. Among the genes, the expression levels of *OsIRT1*, *OsIRT2*, *OsZIP5*, *OsZIP6* and *OsZIP9* were upregulated by Cd in both the WT and *OsLCT2* overexpression lines (Fig. 6). The expression levels of most *OsZIP* genes did not differ between the *OsLCT2* overexpression and WT plants, with the exception of *OsZIP9* expression, which was significantly higher in the overexpression lines than in the WT, irrespective of Cd stress (Fig. 6). *Os*ZIP9 was recently reported to play an important role in the uptake of Zn in rice (Huang et al. 2020; Tan et al. 2020; Yang et al. 2020), so the increased concentrations of Zn in roots of overexpression lines may have been caused by the up-regulation of *OsZIP9* expression.

## Discussion

### OsLCT2 is a Novel LCT Transporter in Rice

To date, rice OsLCT1 and wheat TaLCT1 are the only two LCT members that have been reported in the LCT family. In this study, we characterized OsLCT2 as a new member of the LCT family. Like OsLCT1 and TaLCT1, OsLCT2 is a typical transmembrane protein harboring PEST sequences at the N terminus (Fig. 1b). Previous reports demonstrated that TaLCT1 exhibited influx activity of Cd, Ca, K and Na (Kennedy and Rinne 1997; Clemens et al. 1998), whereas OsLCT1 exhibited efflux activity of Cd, Mg, Ca, Mn and K (Uraguchi et al. 2011, 2014). Likewise, OsLCT2 may have broad substrate specificity in transporting Cd, Mn, Fe and Zn (Fig. 5).

We determined that OsLCT2 and OsLCT1 have 33% sequence identity and belong to different subgroups of the LCT family (Fig. 1b, c). In the Nipponbare genome, *OsLCT2* is absent, whereas *OsLCT1* is located at position 22,566,775–22,571,982 on chromosome 6. We aligned the region ranging from 20.0 Mb to 24.0 Mb on chromosome 6 of Nipponbare to Shuhui498 genome. As a result, we found that the 0.5 M interval (from 22.5 to 23.0 Mb) encompassing *OsLCT1* on chromosome 6 of Nipponbare had extensive sequence divergence from the corresponding interval containing *OsLCT2* on chromosome 6 of Shuhui498 (Additional file 1: Fig. S4). The results of the genome alignment suggested that *OsLCT1* and *OsLCT2* were located in the same genomic region of chromosome 6, but there were large fragments variation in this region, resulting in the low sequence similarity observed between *OsLCT1* and *OsLCT2*. Although *OsLCT2* had 9 haplotypes, haplotype 1 in Huazhan and Shuhui498 was the only major allele, whereas other alleles were rare (Additional file 1: Fig. S1b, c). Moreover, *OsLCT2* was absent from the genomes of some rice varieties. Compared with *indica* and *tropical japonica*, *temperate japonica* and *aus* rice exhibited much higher incidence of *OsLCT2* deletion. (Additional file 1: Fig. S1b, c). In these rice varieties lacking *OsLCT2, OsLCT1* might be present in their genomes.

TaLCT1 is the closest homolog of OsLCT2, and they both belong to the same subgroup with 55% sequence identity (Fig. 1b, c). OsLCT1 is localized to the plasma membrane and its transcripts are mainly found in nodes and leaf blades during the grain-ripening stage, playing an important role in regulation of Cd loading to the phloem and Cd concentrations in grains of rice (Uraguchi et al. 2011). *TaLCT1* is expressed in wheat roots and leaves (Kennedy and Rinne 1997). However, no study has investigated the phenotypes of *TaLCT1* transgenic lines, so its function *in planta* is unclear. *OsLCT2* is also expressed in roots and leaves (Fig. 2a), and this protein might be localized to the ER (Fig. 3). So *OsLCT2* has completely distinct features in amino acid sequences, expression pattern, and subcellular localization from those of *OsLCT1*.

### Overexpression of OsLCT2 Reduces xylem-Mediated Cd Translocation from Roots to Shoots

Root-to-shoot translocation via the xylem is considered as a critical process determining the accumulation of Cd in shoots and grains of rice (Uraguchi et al. 2009). Therefore, it is essential to study the factors controlling Cd translocation to shoots in order to minimize Cd accumulation in grains. The total amount of Cd transported by this process depends mainly on two aspects, one is the efficiency of xylem loading and velocity of xylem transport, and the other is the ability of roots to retain Cd. In roots, Cd appears to use the Zn- and Ca-transport systems to translocate to shoots. The Zn transporters OsHMA2 and OsZIP7 and Ca transporter OsCCX2 have been shown to mediate root-to-shoot translocation of Cd (Satoh-Nagasawa et al. 2012; Takahashi et al. 2012; Yamaji et al. 2013; Hao et al. 2018; Tan et al. 2019). OsHMA3 has been the only transporter identified to inhibit root-to-shoot Cd translocation by vacuolar sequestration of Cd in roots (Ueno et al. 2010). OsHMA3 is also responsible for both Zn detoxification and storage by vacuolar sequestration of Zn (Cai et al. 2019). When overexpressed, *OsHMA3* leads to more Zn sequestered into the vacuoles of root cells but does not affect the Zn accumulated in shoots and grains through up-regulating expression of *OsZIP* genes encoding transporters implicated in uptake, translocation and distribution of Zn (Sasaki et al. 2014; Ueno et al. 2010; Lu et al. 2019; Chen et al. 2021).

Here, overexpression of *OsLCT2* enhances tolerance to excess Cd and reduces Cd accumulation in rice shoots and grains by limiting the amounts of Cd loaded into the xylem for root-to-shoot translocation. *OsLCT2* was expressed in all tissues of the elongation and maturation zones of roots (Fig. 2d). These results raise a possibility that *OsLCT2* overexpression restricts the radial movement of Cd into the xylem and subsequent translocation to the above-ground tissues by retaining a portion of Cd in the ER of root cells. We conducted heterologous expression of *OsLCT2* in yeast. However, yeast could not grow when *OsLCT2* was expressed in it, suggesting that expression of *OsLCT2* has a lethal effect on yeast. The transport activity of OsLCT2 remains to be fully elucidated.

### Overexpression of *OsLCT2* May affect Zn, Fe and Mn Accumulation in Rice

Excessive expression of *OsLCT2* also affected the root-to-shoot translocation of Mn, Fe and Zn, but not of Cu, Ca and Mg. This is supported by our evidence that the hydroponically-grown overexpression lines exhibited lower Mn, Fe and Zn concentrations in shoots and xylem sap and lower rates of root-to-shoot translocation of these metals (Fig. 5f, k, l; Additional file 1: Fig. S3a–c), but similar Cu, Ca and Mg concentrations in shoots and roots compared to those of WT plants (Fig. 5e–h). However, overexpressing *OsLCT2* did not affect the accumulation of Zn, Fe and Mn in straw (Fig. 4g–i) and grains (Fig. 4l–n) at the maturity period of rice in field conditions. These results suggest that the effect of *OsLCT2* overexpression on accumulation of Mn, Fe and Zn is partially dependent on the environment or plant growth stage. We speculate the principal reason for this inconsistent performance of overexpression lines cultivated in nutrient solutions and field soils might be the differences of culture environments**.**

As essential micronutrients, Mn, Fe and Zn are required for many biological processes, but excessive Mn, Fe and Zn are toxic to plants (Bashir et al. 2019). Hence, it is likely that rice has evolutionarily developed efficient sensing systems and complex regulatory networks to maintain homeostasis of these metals. In the hydroponic condition, the concentrations of Mn, Fe and Zn in the nutrient solution were easily controlled, and therefore supplies of these micronutrients were constant and sufficient for plant growth. Although *OsLCT2* overexpression caused decreases in concentrations of Mn, Fe and Zn in shoots (Fig. 5f), the amounts in these plants were sufficient for the normal growth and development (Fig. 5a). So it was likely that the signals of Mn, Fe, or Zn deficiency in the overexpression lines were too weak to induce adequate changes in expression of other metal transporters to offset the effect of *OsLCT2* overexpression on reduction of metal concentrations in shoots. This was supported by our observation that the induction of *OsZIP9* expression activated the uptake of Zn, which increased the concentration of Zn in roots; however, the up-regulation of *OsZIP9* expression was not enough to overcome the decrease of Zn concentration in shoots caused by *OsLCT2* overexpression (Fig. 5f).

However, in field conditions, the contents of Mn, Fe and Zn in the soils fluctuated greatly and frequently, so metal ion homeostasis in plants overexpressing *OsLCT2* was likely disturbed to a greater degree than in hydroponically cultivated plants overexpressing *OsLCT2*. Therefore, the transcription or protein levels of transporters implicated in uptake and translocation of Mn, Fe and Zn may be regulated in field-grown plants to a greater extent to compensate for the lower Mn, Fe and Zn concentrations in shoots resulting from *OsLCT2* overexpression. Similarly, the overexpression of *OsZIP9* also led to inconsistent Zn accumulation characteristics in rice seedlings growing under hydroponic conditions and in mature rice growing in field conditions (Yang et al. 2020).

We also performed hydroponic culture for *OsLCT2* knockout lines and WT plants, treating them with 0.5 μM Cd for two weeks and determined metal concentrations. The concentrations of Cd, Mn, Zn, Fe, and Cu in shoots and roots were similar between the knockout lines and WT plants (Additional file 2: Table S1). The lack of an obvious phenotype in the *OsLCT2* knockout lines may be a result of the low expression level of *OsLCT2* (Additional file 1: Fig. S5).

The localization of OsLCT2 to the ER when its coding gene was driven by 35S promoter suggests that *OsLCT2* overexpression might affect the distribution and homeostasis of Zn, Mn and Fe in cells. Moreover, its overexpression was prone to reduce grain yield, which might be attributed to the higher sensitivity of reproductive cells to the disrupted homeostasis of intracellular metal. This also indicates that the promotion of *OsLCT2* expression by a strong constitutive promoter is not an ideal strategy for breeding rice cultivars accumulating low levels of Cd. Instead, enhancing expression of *OsLCT2* by a root-specific promoter could be a better strategy for breeding rice cultivars with low Cd concentrations in rice grains.

## Conclusions

A member of the low-affinity cation transporter (*LCT*) family, *OsLCT2* was cloned and characterized from rice. *OsLCT2* was expressed in all tissues of the elongation and maturation zones in roots. Overexpression of *OsLCT2* significantly reduced Cd concentrations in the shoots and grains by weakening xylem-mediated Cd translocation from roots to shoots in rice. Furthermore, its overexpression induced the expression of *OsZIP9*, a gene responsible for Zn uptake, and consequently increased Zn concentrations in roots. Our findings provide a new genetic resource to be manipulated to reduce Cd level in rice grains.

## Methods

### Plant Material and Growth Conditions

An *indica* cultivar Huazhan was used to determine *OsLCT2* expression patterns and as the WT rice for phenotypic analyses. *OsLCT2* overexpression lines, knockout lines and *OsLCT2*-promoter::*GUS* plants were generated in the background of cv. Huazhan.

Rice seedlings were grown hydroponically for *OsLCT2* expression analysis and phenotypic analysis of overexpression plants. Rice seeds were soaked in tap water for 2 d and allowed 1 d for germination at 37℃. Those seeds that germinated were sown into a floating plate with holes and placed on distilled water. After 7 d, seedlings were transferred to plastic pots containing Yoshida nutrient solution as described previously (Yoshida et al. 1976). The pH of the nutrient solution was adjusted to 5.5 and renewed every 3 d. Plants were grown in a growth chamber with a 14-h light (28 °C)/10-h dark (24 °C) photoperiod, 700 μmol m^−1^ s^−1^ light intensity and 60–70% humidity.

*OsLCT2*-overexpressing lines, knockout lines and the WT were grown at a Cd-contaminated experimental paddy field in Hunan Province of China to evaluate their agronomic and grain ionomic traits. The soil Cd concentration was approximately 0.65 mg/kg and soil pH was 5.8. Rice seeds were sown on the seed bed and grown for 26 days, and then the seedlings were transplanted into the field, spaced at 17 cm × 20 cm. The field experiments were arranged in a randomized complete block design with three replications. The plants of each line were grown in 4 rows × 8 plants in each plot. The fields were irrigated intermittently until the rice grains ripened.

### Cloning of *OsLCT2* and Phylogenetic Analysis

To obtain the full-length cDNA sequence of *OsLCT2*, total RNA was extracted from rice roots (cv. Huazhan) using the Plant RNA Kit (Omega), and the full-length cDNA sequence of *OsLCT2* was achieved by RACE using the SMARTer® RACE 5′/3′ Kit (Clontech). The open reading frame of *OsLCT2* was amplified by RT-PCR using specific primers (Additional file 2: Table S2) designed according to the sequence information obtained from the full-length *OsLCT2* cDNA.

To perform a phylogenetic analysis, BLAST searches were performed to extract amino acid sequences of LCT-like proteins in different plant species from the NCBI (http://www.ncbi.nlm.nih.gov/) databases. LCT-like proteins in wild rice species were detected by BLAST searches of the UniProt database (https://www.uniprot.org/). The amino acid sequences were aligned using the ClustalW program, and the phylogenetic tree was constructed using MEGA7 (http://www.megasoftware.net/) by the neighbor-joining method with 1000 bootstrap trials. Trans-membrane domains were predicted with both the TMPRED program (https://embnet.vital-it.ch/software/TMPRED_form.html/) and the CCTOP program (http://cctop.enzim.ttk.mta.hu/). Genome sequence alignment was performed using MUMmer 4 (http://mummer.sourceforge.net/). The haplotypes of *OsLCT2* in the 3620 rice accessions were classified according to the SNPs within the CDS region using the MBKbase-rice database (http://www.mbkbase.org/rice/).

### Construction and Transformation of Plant Expression Vectors

To generate the *OsLCT2*-promoter::*GUS* vector, the 2167-bp promoter sequence, upstream of the start codon, was amplified by PCR from the genomic DNA of rice cv. Huazhan and cloned into a *Pst* I/*Nco* I-digested pCAMBIA1301 vector using the CloneExpress® II One Step Cloning Kit (Vazyme). To generate the overexpression vector of *OsLCT2*, the ORF of *OsLCT2* was amplified by PCR from the total cDNA of Huazhan and cloned into the *Kpn* I/*Spe* I-digested pTCK303 vector under the control of the ubiquitin promoter using the same recombination cloning method described above. The PCR primers are listed in Additional file 2: Table S2. A knockout construct of *OsLCT2* was generated by CRISPR/Cas9 technology (Ma and Liu 2016), targeting two sequences at the third exon of *OsLCT2* (Fig. 4b). All constructs were transformed into Huazhan using *Agrobacterium*-mediated transformation, which was conducted by Wuhan Biorun Biological Technology Co., Ltd. The homozygous overexpression lines were selected by hygromycin B screening and expression analysis. The homozygous knockout lines without *Cas9* were selected by PCR and DNA sequencing.

### Gene Expression Analysis

Leaves, roots and the basal region (2 cm above the roots) of rice seedlings (14 d old) grown in Yoshida nutrient solution were sampled for expression pattern analysis. The tissue of the seedlings with the highest *OsLCT2* expression level was selected for use in the subsequent metal-response analyses. *OsLCT2* expression in response to metal excess or deficiency was determined by exposing the one-week-old Huazhan seedlings to the Yoshida nutrient solutions without Fe, Mn, or Zn, or with high concentrations of Fe (500 or 1000 μM FeSO_4_, pH 4.5), Mn (100 or 500 μM MnCl_2_), or Zn (40 or 100 μM ZnSO_4_) for 7 d, based on previous reports (Lee et al. 2007; Sasaki et al. 2011; Aung et al. 2018; Liu et al. 2019; Tan et al. 2020). The levels of metal deficiency in rice were determined using the appropriate marker genes (Zheng et al. 2012). To determine *OsLCT2* expression in response to Cd, two-week-old Huazhan seedlings were exposed to 0.5, 2.5 or 25 μM CdCl_2_ for 24 h. Seedlings grown in standard Yoshida nutrient solution were used as controls. After Cd treatments, leaves, roots and basal regions were separated for RNA extraction. The *OsLCT2* overexpression lines and WT plants were used to analyze the effect of *OsLCT2* overexpression on the expression of *OsZIP* genes. Two-week-old rice seedlings grown hydroponically were exposed to 0 or 0.5 μM CdCl_2_ for 1 d, and the roots were sampled for RNA extraction.

The total RNA of samples was extracted and then converted to cDNA using the PrimeScript™ RT reagent kit with gDNA Eraser (Takara). qRT-PCR was conducted using TB Green® Premix Ex Taq™ II (TaKaRa) with a LightCycler 480II PCR instrument (Roche). The expression level of *OsLCT2* and ten *OsZIP* genes were determined. *Histone H3* and *Actin1* were used as internal controls. All primers used for qRT-PCR are listed in Additional file 2: Table S2.

### GUS Histochemical Analysis

A GUS histochemical analysis was performed on transgenic *OsLCT2*-promoter::*GUS* plants. The transgenic plant samples were soaked in fixation buffer for 45 min and then incubated in X-Gluc staining buffer at 37 °C overnight using GUS Stain Kit (Solarbio, G3060) following the manufacturer’s instructions. The stained samples were held in 70% (v/v) ethanol and observed under a stereomicroscope (Smart Zoom 5, Zeiss). To make paraffin sections, stained tissues were fixed in FAA (formalin-acetic acid-50% ethanol [1:1:18]), vacuum-infiltrated for 10 min, then kept for 24 h. The fixed samples were dehydrated in a graded ethanol series (50%, 60%, 70%, 80%, 90%, 95%, 100%) and cleared in a gradient xylene series (50%, 75%, 90%, 100%). The samples were then embedded in paraffin, sectioned to 8 μm thickness using a microtome (HM340E, Thermo) and photographed under a microscope (Leica).

### Subcellular Localization Analysis

A 16 amino-acid (aa) linker was synthesized and inserted into the pAN580 vector between *Nhe* I and *Bam*H I restriction sites at the N-terminus of eGFP in the pAN580 vector. The ORF sequence of *OsLCT2* without the stop codon was amplified using total cDNA of Huazhan. The PCR product was then cloned into the *Spe* I/*Nhe* I-digested pAN580 vector with the linker to generate the *35S::OsLCT2-linker-eGFP* vector. The 16 aa linker was also inserted into the pAN580 vector between *Bgl* II and *Pst* I restriction sites fused in-frame to the C-terminus of eGFP. The ORF sequence of *OsLCT2* was cloned into the *Pst* I/*Eco*R I-digested pAN580 vector with the linker to generate the *35S::eGFP-linker-OsLCT2* vector. These vectors were constructed by recombination using the primers shown in Additional file 2: Table S2 with ClonExpress® II One Step Cloning Kit (Vazyme). The pAN580 vector containing the expression cassette of *35S::eGFP* was used as a control. The *35S::OsLCT2-linker-eGFP*, *35S:: eGFP-linker-OsLCT2* or *35S:: eGFP* construct was co-transformed with vector carrying *35S::HDEL-mCherry* into rice protoplasts via polyethylene glycol-mediated transformation. All protoplasts were observed and imaged using a confocal laser scanning microscope (LSM 880, Zeiss) after incubation at 28 °C for 16 to 24 h.

### Determination of Metal Concentrations in Plant Tissues

Two-week-old seedlings of hydroponically-grown rice were exposed to a nutrient solution with either 0, 0.5 or 1 μM CdCl_2_ for 14 days. Then the samples were washed with distilled water three times and separated into shoots and roots. Field-grown rice plants were harvested after grain ripening and separated into straw (shoot) and brown rice (grain).

All samples were dried at 70 °C for 3 d, and then digested with an acid mixture of HNO_3_:HClO_4_ (6:1 [v/v]) as described previously (Tang et al. 2017). The metal concentrations in the digest solutions were determined by inductively coupled plasma mass spectrometry (ICP-MS) (Agilent 7700 series, USA). The root-to-shoot translocation rates of metals were calculated as the amount of metal accumulated in the shoots as a percentage of the total amount of metal accumulated in the whole plant, as described by Miyadate et al. (2011).

### Collection and Analysis of xylem Sap

After two weeks of Cd treatment, the 4-week-old seedlings of two *OsLCT2*-overexpressing and WT plants were cut with a razor at about 2 cm above the root-shoot junction. Xylem sap was collected from the cut surface for 1 h using micropipettes. The first drop of xylem sap emerging from the cut end was discarded to avoid contamination from the symplastic metal ions in the cells damaged by cutting. Xylem sap from 20 plants was combined as one biological replicate and three biological replicates were made. Metal concentrations in the xylem sap were determined by ICP-MS.

## Supplementary Information

**Additional file 1.** **Supplemental Figures. Fig. S1.** Genetic diversity of *OsLCT2*. **Fig. S2.** Agronomic traits of *OsLCT2* overexpression lines and the WT grown in paddy fields. **Fig. S3.** Metal concentrations in shoots and roots of overexpression lines and the WT treated with Cd for 14 days. **Fig. S4.** Synteny analysis of regions encompassing *OsLCT1* or *OsLCT2* on chromosome 6 between *O. sativa* cv. Nipponbare and cv. Shuhui498. **Fig. S5.** qRT-PCR-based expression analysis of *OsLCT2* in cv. Huazhan.

**Additional file 2.** **Supplemental Tables. Table S1.** Concentrations of metal in shoots and roots of *OsLCT2* seedling (mg/kg). **Table S2.** Primers used in the present study.

## Data Availability

The datasets supporting the conclusions of this article are included within the article and its additional files.

## Abbreviations

Cd: Cadmium
Mn: Manganese
Fe: Iron
Zn: Zinc
Ca: Calcium
K: Potassium
Mg: Magnesium
LCT: Low-affinity cation transporter
NRAMP: Natural resistance-associated macrophage protein
ZIP: Zinc-regulated/iron-regulated transporter-like protein
HMA: Heavy metal ATPase
ER: Endoplasmic reticulum
NCBI: National center for biotechnology information
RACE: Rapid amplification of cDNA ends
ORF: Open reading frame
GUS: β-Glucuronidase
HDEL: His-Asp-Glu-Leu
qRT-PCR: Quantitative real-time PCR
eGFP: Enhanced green fluorescent protein
SNPs: Single-nucleotide polymorphisms
