# Supplementary material for: Overexpression of OsLCT2, a Low-Affinity Cation Transporter Gene, Reduces Cadmium Accumulation in Shoots and Grains of Rice
Source: Rice (N Y). 2021 Oct 24;14:89. doi: 10.1186/s12284-021-00530-8 (PMC8542528; doi:10.1186/s12284-021-00530-8)
Supplement: Supplementary file 1 — Additional file 1. Supplemental Figures. Fig. S1. Genetic diversity of OsLCT2. Fig. S2. Agronomic traits of OsLCT2 overexpression lines and the WT grown in paddy fields. Fig. S3. Metal concentrations in shoots and roots of overexpression lines and the WT treated with Cd for 14 days. Fig. S4. Synteny analysis of regions encompassing OsLCT1 or OsLCT2 on chromosome 6 between O. sativa cv. Nipponbare and cv. Shuhui498. Fig. S5. qRT-PCR-based expression analysis of OsLCT2 in cv. Huazhan. [file 12284_2021_530_MOESM1_ESM.docx]

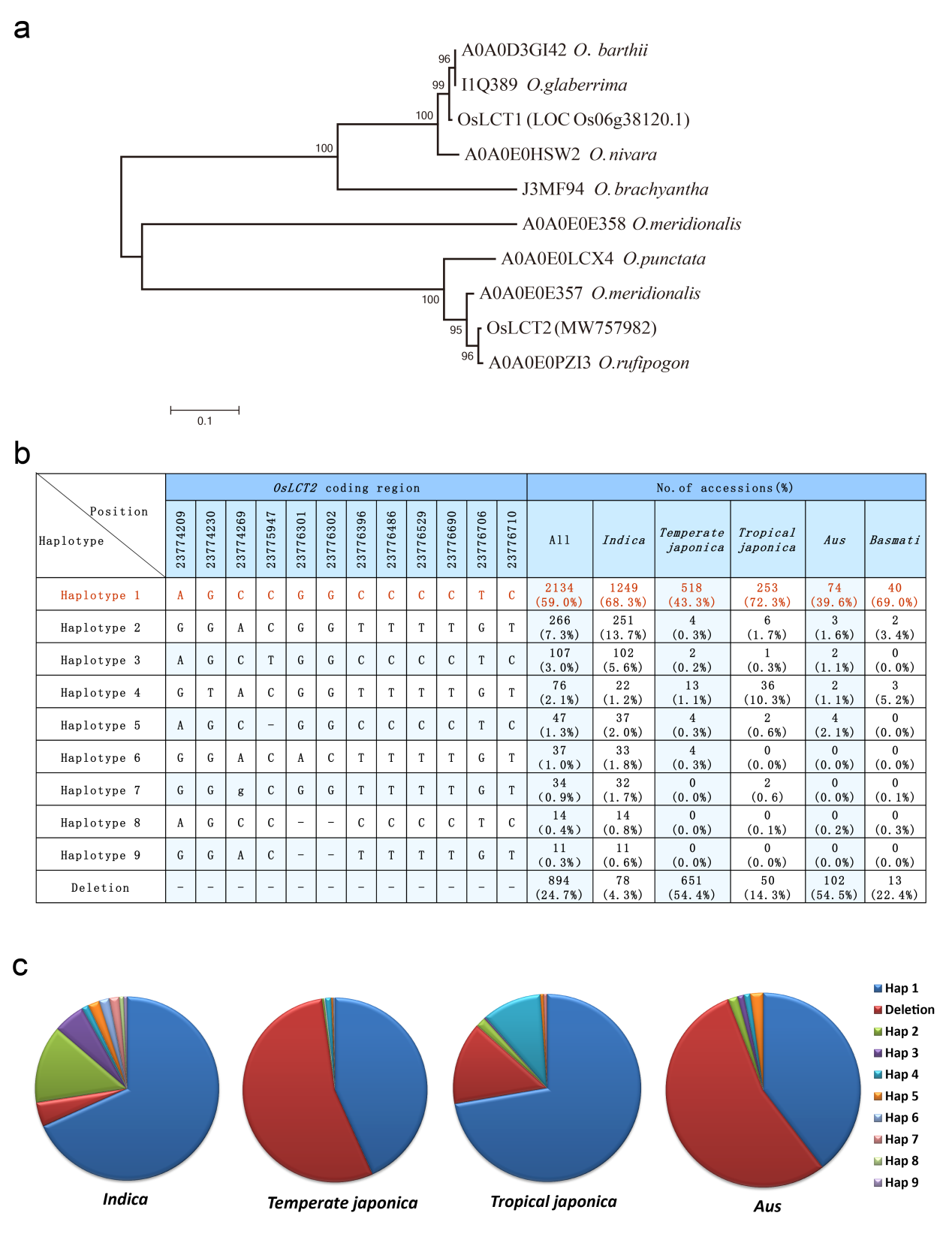


**Fig. S1** Genetic diversity of *OsLCT2*. (a**)** Phylogenic analysis of OsLCT1, OsLCT2 and their homologs in wild *Oryza* species. The phylogenetic tree was constructed by MEGA 7 using the neighbor-joining method. The scale bar of 0.1 represents 10% sequence divergence. (b) Haplotype analysis of *OsLCT2* coding regions from 3,620 rice accessions of the MBKbase-rice database (rare haplotypes of <10 accessions are not shown). Lowercase letter represents synonymous mutation, whereas uppercase letter indicates non-synonymous mutation. Dash line indicates the base is absent. Number and percentage of each haplotype in a given subpopulation are shown. (c) Distribution frequency of haplotypes and deletions of *OsLCT2* in different rice subpopulations.

**Fig. S2** Agronomic traits of *OsLCT2* overexpression lines and the WT grown in paddy fields. (a) Plant height. (b) Effective tilling number. (c) Total grain number per panicle. (d) Filled grain number per panicle. (e) Seed setting rate. (f) 1000-grain weight. Data are means ± SD of three biological replicates. Asterisks above the bars indicate significant differences from the WT (* *P* < 0.05, ** *P* < 0.01; Student’s *t*-test).

**Fig. S3** Metal concentrations in shoots and roots of overexpression lines and the WT treated with Cd for 14 days. (a-c) Mn (a), Fe (b), Zn (c) concentrations in the shoots of overexpression lines and the WT at 0.5 or 1 μM Cd supply. (d-f) Mn (d), Fe (e), Zn (f) concentrations in the roots of overexpression lines and the WT at 0.5 or 1 μM Cd supply. Asterisks above the bars indicate significant differences from the WT (* *P* < 0.05, ** *P* < 0.01; Student’s *t*-test).


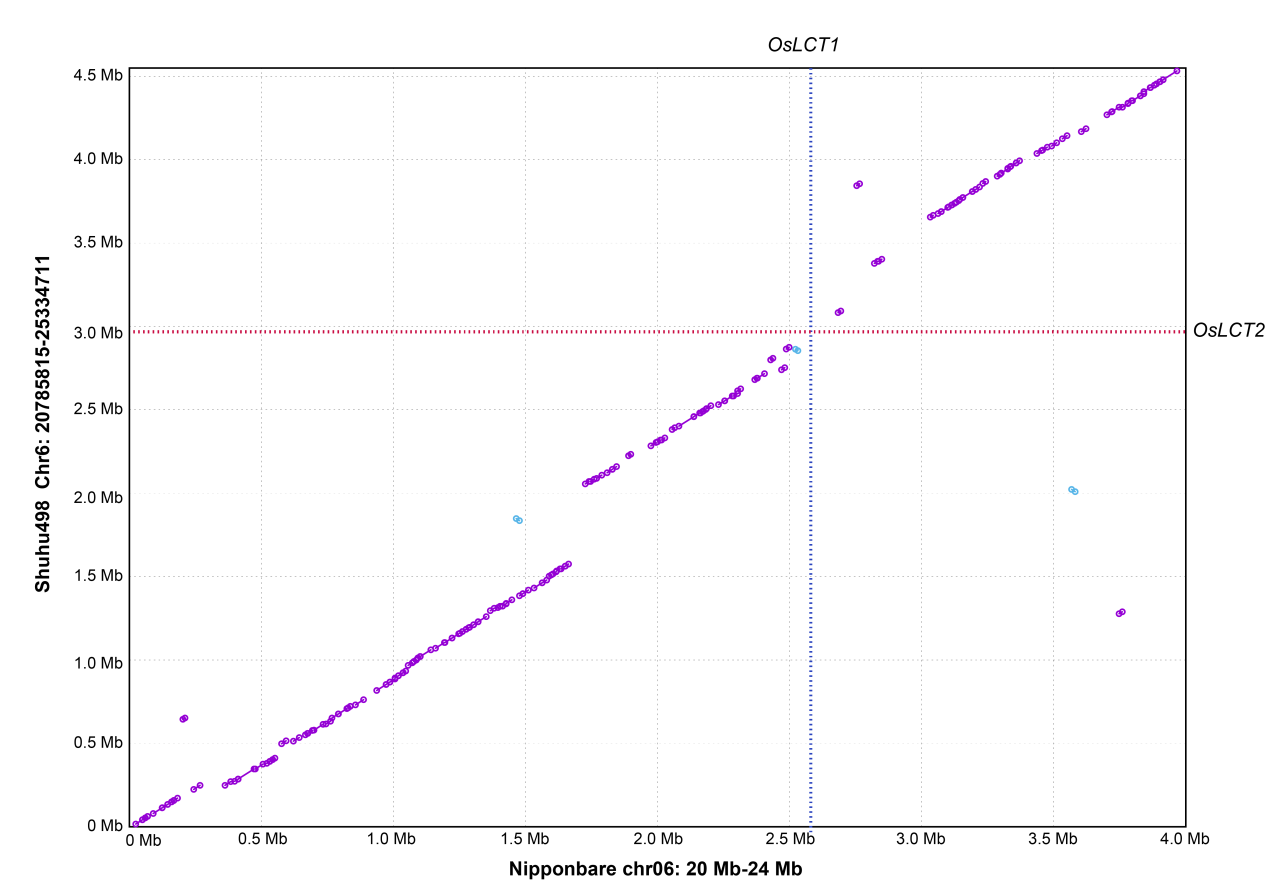


**Fig. S4** Synteny analysis of regions encompassing *OsLCT1* or *OsLCT2* on chromosome 6 between *O. sativa* cv. Nipponbare and cv. Shuhui498. Purple dots and lines represent collinear homologs. The blue dotted line shows the approximate position of *OsLCT1*. The red dotted line indicates the approximate position of *OsLCT2*.

**Fig. S5** qRT-PCR-based expression analysis of *OsLCT2* in cv. Huazhan. The leaf, root and basal stem (2 cm above the roots) in 2-week-old seedlings grown hydroponically were sampled for expression analysis. Expression levels of *OsLCT2* relative to the internal reference gene *OsActin1* are shown as means ± SD of three biological replicates.
