# Supplementary material for: Overexpression of OsLCT2, a Low-Affinity Cation Transporter Gene, Reduces Cadmium Accumulation in Shoots and Grains of Rice
Source: Rice (N Y). 2021 Oct 24;14:89. doi: 10.1186/s12284-021-00530-8 (PMC8542528; doi:10.1186/s12284-021-00530-8)
Supplement: Supplementary file 2 — Additional file 2. Supplemental Tables. Table S1. Concentrations of metal in shoots and roots of OsLCT2 seedling (mg/kg). Table S2. Primers used in the present study. [file 12284_2021_530_MOESM2_ESM.docx]

**Table S1 Concentrations of Metal in shoots and roots of *oslct2* seedling (mg/kg)**

|  | Line | Cd | Mn | Zn | Fe | Cu |  |
| --- | --- | --- | --- | --- | --- | --- | --- |
| Shoot | WT | 32.03±1.52 | 825.23±42.06 | 87.70±11.15 | 349.00±32.19 | 17.01±0.53 |  |
|  | T2-KO-13 | 29.38±1.85 | 775.60±36.20 | 82.00±3.12 | 287.67±35.73 | 16.46±0.68 |  |
|  | T2-KO-16 | 31.88±1.51 | 842.57±58.49 | 85.83±12.56 | 307.33±25.11 | 17.32±0.70 |  |
| Root | WT | 236.82±15.95 | 79.60±4.95 | 145.00±7.70 | 6761.05±558.58 | 28.94±2.94 |  |
|  | T2-KO-13 | 244.74±12.47 | 83.30±5.34 | 146.87±7.60 | 6750.33±562.75 | 27.20±0.44 |  |
|  | T2-KO-16 | 250.01±11.00 | 80.03±4.86 | 145.97±8.70 | 6676.12±520.16 | 31.40±3.47 |  |

Note: There is no significant difference on 0.05 level of *P* by Student’s *t* test.

**Table S2. Primers used in the present study**

| **Primer name** | **Sequence(5’-3’)** | **Purpose** |
| --- | --- | --- |
| T2-ORF-F | ATGGCCAACGAGCTCAGCGC | Cloning of *OsLCT2* ORF |
| T2-ORF-R | TCAGATTCTCCGTGCATCCA |  |
| P1301-T2PRO-F | tcctctagagtcgacctgcagCCAGGCTCCAGCCGGCTG | Construction of *OsLCT2*-promoter::*GUS* vector |
| P1301-T2PRO-R | ttaccctcagatctaccatggGGCGCTAGCTCCTCGCTAG |  |
| P303-T2ORF-F | tctagaggatccccgggtaccATGGCCAACGAGCTCAGCG | Construction of *OsLCT2* overexpression vector |
| P303-T2ORF-R | ttcgagctctctagaactagtTCAGATTCTCCGTGCATCCAC |  |
| T2-qPCR-F | TGATGACGCTGTCGAAGAAG | qRT-PCR for *OsLCT2* |
| T2-qPCR-R | GAGCGAGCAGAGCAAGAA |  |
| HIS3-qPCR-F | GGTCAACTTGTTGATTCCCCTCT | qRT-PCR for *OsHistone H3* |
| HIS3-qPCR-R | AACCGCAAAATCCAAAGAACG |  |
| Actin-qPCR-F | GACTCTGGTGATGGTGTCAGC | qRT-PCR for *OsActin1* |
| Actin-qPCR-R | GGCTGGAAGAGGACCTCAGG |  |
| OsIRT1-qPCR-F | GGCGATGTGCTTCCACCAGA | qRT-PCR for *OsIRT1* |
| OsIRT1-qPCR-R | GACGAGCACCGACCTCATCC |  |
| OsIRT2-qPCR-F | GTCGAGGCCGGTAACACCAC | qRT-PCR for *OsIRT2* |
| OsIRT2-qPCR-R | TGCACCACGATGCCCATCTC |  |
| OsZIP1-qPCR-F | CGTCATGGCTGTCGTCATGATCTG | qRT-PCR for *OsZIP1* |
| OsZIP1-qPCR-R | AATGGGGTGATAGAAATCGAACATG |  |
| OsZIP2-qPCR-F | TTTCGGACGTTTGTTGGTTC | qRT-PCR for *OsZIP2* |
| OsZIP2-qPCR-R | TCCTGAAACTTTGGTTGGAGT |  |
| OsZIP4-qPCR-F | CACCAGTTCTTTGAAGGCATTG | qRT-PCR for *OsZIP4* |
| OsZIP4-qPCR-R | AGCTGGATGAGATTGCGATC |  |
| OsZIP5-qPCR-F | CATGAAGACCAAGGTGCAGAGAAGG | qRT-PCR for *OsZIP5* |
| OsZIP5-qPCR-R | TCACGCCCAGATGGCGATCA |  |
| OsZIP6-qPCR-F | TCTACATGGCACTTGTCGATCTC | qRT-PCR for *OsZIP6* |
| OsZIP6-qPCR-R | GACATGGATGCAGATCCAAGCA |  |
| OsZIP7-qPCR-F | CCTTGCAATCTGGGCCTGAA | qRT-PCR for *OsZIP7* |
| OsZIP7-qPCR-R | CAGATTAGTCTCACGCCCATGA |  |
| OsZIP8-qPCR-F | GGTGCAGAGCAAAGGCAAGCT | qRT-PCR for *OsZIP8* |
| OsZIP8-qPCR-R | AATTTCCTCTACATTAGTCCCTGA |  |
| OsZIP9-qPCR-F | ATCTTCTTCTCGCTAACCACAC | qRT-PCR for *OsZIP9* |
| OsZIP9-qPCR-R | GCAGCCGCTGCGTCGAGAAT |  |
| P580-T2CDS-F | ggacagcccagatcaactagtATGGCCAACGAGCTCAGCG | Construction of *35S::OsLCT2-eGFP* vector |
| P580-T2CDS-R | gcccttgctcaccatggatccGATTCTCCGTGCATCCACACC |  |
| Linker | TCAGGTGGAGGCGGTTCAGGCGGAGGTGGCTCTGGCGGTGGCGGATCG | Construction of vector for subcellular localization |
| P580-T2-GFP-F | ggacagcccagatcaactagtATGGCCAACGAGCTCAGCG | Construction of *35S::OsLCT2-linker-eGFP* vector |
| P580- T2-GFP-R | gcctgaaccgcctccacctgaGATTCTCCGTGCATCCACACC |  |
| P580- GFP-T2-F | ggctctggcggtggcggatcgATGGCCAACGAGCTCAGCG | Construction of *35S:: eGFP-linker-OsLCT2* vector |
| P580- GFP-T2--R | gataagcttgatatcgaattcTCAGATTCTCCGTGCATCCAC |  |
